# Diversity in clinical management and protocols for the treatment of major bleeding trauma patients across European level I Trauma Centres

**DOI:** 10.1186/s13049-015-0147-6

**Published:** 2015-10-01

**Authors:** Nadine Schäfer, Arne Driessen, Matthias Fröhlich, Ewa K. Stürmer, Marc Maegele, TACTIC partners

**Affiliations:** Institute for Research in Operative Medicine (IFOM), Witten/Herdecke University (Campus Cologne-Merheim), Ostmerheimerstr. 200, 51109 Cologne, Germany; Department of Orthopaedic Surgery, Traumatology and Sports Traumatology, Cologne-Merheim Medical Center (CMMC), Witten/Herdecke University (Campus Cologne-Merheim), Ostmerheimerstr. 200, 51109 Cologne, Germany

## Abstract

**Background:**

Uncontrolled haemorrhage is still the leading cause of preventable death after trauma and the primary focus of any treatment strategy should be related to early detection and control of blood loss including haemostasis.

**Methods:**

For assessing management practices across six European level I trauma centres with academic interest and research in the field of coagulopathy an online survey was conducted addressing local management practice for bleeding trauma patients including algorithms for detection, management and monitoring coagulation disorders and immediate interventions. Each centre provided their locally applied massive transfusion protocol.

**Results:**

All participating trauma centres have developed and implemented a local algorithm and protocol for the bleeding trauma patient. These are uniformly activated by clinical triggers and deactivated once the bleeding has stopped according to clinical assessment in combination with laboratory signs of achieved haemostasis. The severity of coagulopathy and shock is mostly assessed via standard coagulation tests and partially used extended viscoelastic tests. All centres have implemented the immediate use of tranexamic acid. Initial resuscitation is started either pre-hospital or after hospital admission by using transfusion packages with pre-fixed universal blood product combinations and ratios following the concept of “damage control resuscitation” at which applied ratios substantially vary. Two centres initially start with transfusion packages but with viscoelastic tests running in parallel to quickly allow a shift towards a viscoelastic test-guided therapy.

**Conclusion:**

Diversity in the management of bleeding trauma patients such as pre-hospital blood administration and routinely performed viscoelastic tests exists even among level I trauma centres. The paucity of consensus among these centres highlights the need for further primary research followed by clinical trials to improve the evidence for sophisticated guidelines and strategies.

**Electronic supplementary material:**

The online version of this article (doi:10.1186/s13049-015-0147-6) contains supplementary material, which is available to authorized users.

## Introduction

Modern trauma care has demonstrated to lower mortality by creating multidisciplinary evidence-based treatment algorithms for the bleeding trauma patient, by creating awareness among the involved medical specialties and by improving mutual understanding [1, 2]. Several examples have emphasized the value of treatment algorithms in improving trauma care and, vice-versa, deviations from these pathways have increased both morbidity and mortality with a three-fold increased mortality in the subgroup of major deviations [3, 4]. Riskin and colleagues have reported a reduction in mortality from 45 to 19 % after the implementation of a massive transfusion protocol for bleeding trauma patients in their local setting [4]. But despite major improvements in resuscitation that have been integrated into clinical trauma care, uncontrolled haemorrhage is still one of the leading causes of preventable death after injury [5–8]. Therefore, the primary focus of any treatment strategy should be related to the early detection and control of blood loss including haemostasis. The need for an efficient and rapid treatment of patients with uncontrolled bleeding and coagulopathy becomes evident since half of all trauma-related deaths occur within 6 h of hospital admission [9, 10]. One quarter of all severely injured and haemodynamically shocked patients develop clotting abnormalities within minutes of injury. This exacerbates life-threatening bleeding and is associated with dramatically increased morbidity and mortality [11].

Although most facilities have implemented guidelines and protocols to quickly assess and treat trauma patients to date, there is high diversity in clinical practice including diagnosis of major bleeding and trauma-induced coagulopathy [3, 4, 12–16]. These protocols may vary depending upon local infrastructure and logistics, thus, making outcome comparisons between individual centres rather difficult.

The present study aims to describe the current clinical practice management for severely bleeding trauma patients across six central and northern European level I trauma centres with long standing scientific expertise in the field of coagulopathy. All centres are founding members of the International Trauma Research Network (INTRN) and currently run the EU FP-7-funded research project “Targeted Action to Cure Trauma-Induced Coagulopathy (TACTIC)” which aims to deliver universal guidelines for different environments on how to treat haemostatic abnormalities in trauma (http://www.tacticgroup.dk/).

## Material and methods

### Trauma centres

**Fig. 1:**
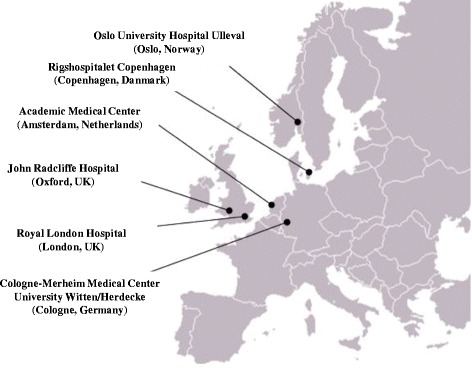
INTRN-affiliated trauma centres across central and northern Europe

The founding trauma centres affiliated to the INTRN that are also involved in the EU FP-7 TACTIC project are the Oslo University Hospital Ulleval (Oslo (OSL), Norway), Rigshospitalet Copenhagen (Copenhagen (CPH), Denmark), Academic Medical Centrum (Amsterdam (AMC), The Netherlands), John Radcliffe Hospital (Oxford (OXF), UK), Royal London Hospital (London (RLH), UK), Cologne Merheim Medical Center, University Witten/Herdecke (Cologne (COL), Germany) (Fig. 1).

As part of a EU FP-7 TACTIC work package (WP 3) the present study aims to describe the current clinical practice management for severely bleeding trauma patients across six central and northern European level I trauma centres who are EU FP-7 TACTIC affiliated partners. The participation of other centres was not taken into account.

### Online survey

An online survey was designed and programmed with accreditation and support of the IT division of the University Witten/Herdecke using the Lime Survey application (http://www.limesurvey.org/). The survey included a set of 13 questions to be answered via single or multiple-choice answers, a drop down menu and one description field for manual entry (Additional file 1). The link to access the questionnaire was distributed via mail to the INTRN-site leads of the centres mentioned above.

### Massive transfusion protocols (MTPs) and algorithms

Each centre was further requested to provide their local treatment algorithms and massive transfusions protocols that are activated for the management of bleeding trauma patients in their local settings.

## Results

### Characterization of the six trauma centres

Trauma centres in London and Oslo receive the greatest number of severely injured patients (ISS ≥16) with more than 400 patients admitted per year, followed by Copenhagen (301–400 patients), Amsterdam and Oxford (201–300 patients), and Cologne (101–200 patients; Table 1). All centres declared that less than 10 % of their patients are considered as severe, coagulopathic bleeders requiring haemostatic therapy including a massive transfusion. Half of the centres reported that pre-hospital blood product administration (CPH, OSL, and OXF) was available. In-hospital rapid administration of blood products, mostly within less than 15 min, is facilitated by an in-house blood bank in all centres. All centres provide multidisciplinary teams for the initial treatment of bleeding trauma patients in the resuscitation room. Almost all reported having immediate access to standard blood products and haemostatic agents including single factor concentrates as shown in Table 2. The availability of cryoprecipitate was restricted to centres in the UK and Copenhagen. Half of the centres reported having thawed plasma for immediate Emergency Department use (AMC, CPH and OXF). Just one centre (OSL) indicated use of Octaplas as Solvent/Detergent treated (virus inactivated) pooled frozen plasma.

**Table 1:** Trauma centre characteristics

Location	Amsterdam	Cologne	Copenhagen	London	Oslo	Oxford
Admitted trauma patients	201–300	100–200	201–300	>400	> 400	201–300
Patients with massive bleeding [%]	< 10	< 10	< 10	< 10	< 10	< 10
Pre-hospital blood administration			x		x	x
Blood bank in-house	x	x	x	x	x	x
Administration first blood product [min]	<15	16–30	<15	<15	<15	<15
Intended transfusion ratio	1:1:1	1:1	1:1:1	1:2	1:1:1	3:2:1
Specialities in the trauma resuscitation room						
General surgery		x		x	x	
Trauma/orthopaedic surgery	x	x	x		x	x
Anaesthesiology	x	x	x	x	x	x
Transfusion medicine			x			
Intensive care/critical care medicine				x		
Accident and emergency medicine	x			x		x
Trauma general surgeons	x				x

**Table 2:** Local availabilities of blood products and haemostatic agents

		No. of centres	%
Blood products	RBCs	6	100
	Platelet concentrates (single platelet units or aphaeresis packs)	6	100
	FFPs	5	83.3
	Cryoprecipitate (FVIII, Fibrinogen, vWF, FXIII)	4	66.7
	Thawed fresh plasma	3	50
	Octaplas	1	16.7
Factor concentrates	Fibrinogen concentrate	5	83.3
	Single factor concentrate (rFVIIa)	5	83.3
	Prothrombin complex concentrate (3- or 4-factor PCC concentrates; PPSB) FII; FVII; FIX; FX, protein C and S	5	83.3
	Single factor concentrate (FXIII)	3	50
	Single factor concentrate (other)	1	16.7
Supportive drugs	Tranexamic acid	6	100
	Calcium (Ca^2+^)	6	100
	Vitamin K	5	83.3
	Aminocaproic acid	1	16.7

### Assessment, diagnosis and monitoring of haemostatic abnormalities/coagulopathy

Table 3 provides an overview of parameters and diagnostic procedures locally in use to assess, diagnose and monitor haemostatic abnormalities and coagulopathy in bleeding trauma patients. The majority of the centres reported using standard laboratory and coagulation tests (haemoglobin (Hb), INR, quantitative fibrinogen and platelet counts) combined with imaging (conventional X-ray ultrasonography and computed tomography) to rapidly assess and monitor haemostatic disorders/coagulopathy. The level of shock is usually determined via point-of-care arterial blood gas (ABG) analysis providing quick information on acid–base status, lactate and pH. Most of the laboratory results are available within 30 min or latest within 1 h, irrespective of centre. Half of all centres or even fewer run functional assays such as viscoelastic tests, aggregometry or functional fibrinogen tests. One centre (COL) assesses patients via a scoring system predicting a massive transfusion that integrates clinical and laboratory findings (e.g. Trauma Associated Severe Haemorrhage (TASH)).

**Table 3:** Diagnostics used to rapidly assess, manage and monitor haemostatic disorders/coagulopathy after trauma

		No. of centres	%
Laboratory tests	Haemoglobin	6	100
	Base excess/deficit (BE/BD)	6	100
	Platelet count	5	83.3
	Fibrinogen (quantitative)	5	83.3
	PT/INR/Quick	5	83.3
	aPTT	4	66.7
	Haematocrit	2	33.3
	Fibrinogen (functional)	2	33.3
	Platelet function (e.g. Aggregometry)	1	16.7
Imaging measure	FAST Ultrasound	6	100
	Imaging (CT)	6	100
Point-of-care diagnostic	Lactate	5	83.3
	pH	5	83.3
	Ionised Calcium	3	50
	Viscoelastic tests (TEG/ROTEM)	3	50
Scoring	Scoring systems (e.g. TASH)	1	16.7

### Local haemostasis, resuscitation and coagulation management in case of major bleeding

**Fig. 2:**
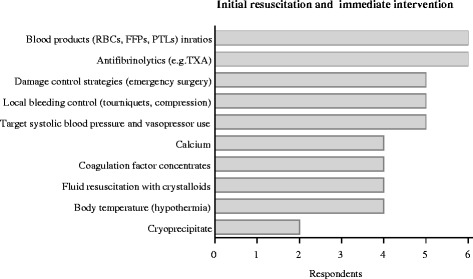
Key measures during initial resuscitation for bleeding control

Key measures in most centres in the acute phase are control of local bleeding sources via tourniquets and compression, the achievement and maintenance of systolic blood pressure via crystalloid volume administration and/or vasopressors, measures to further prevent temperature loss and acidosis and damage control strategies (Fig. 2). All centres recognize the importance of time management in the initial resuscitation of bleeding and coagulopathic trauma patients. For the initial correction of haemostatic abnormalities and coagulopathy, standard blood products, fibrinolysis inhibitor tranexamic acid (TXA), and coagulation factor concentrates either as single factors or in different combinations are immediately available and in use (Table 2).

### Treatment algorithms and massive transfusion protocols (MTPs)

**Fig. 3:**
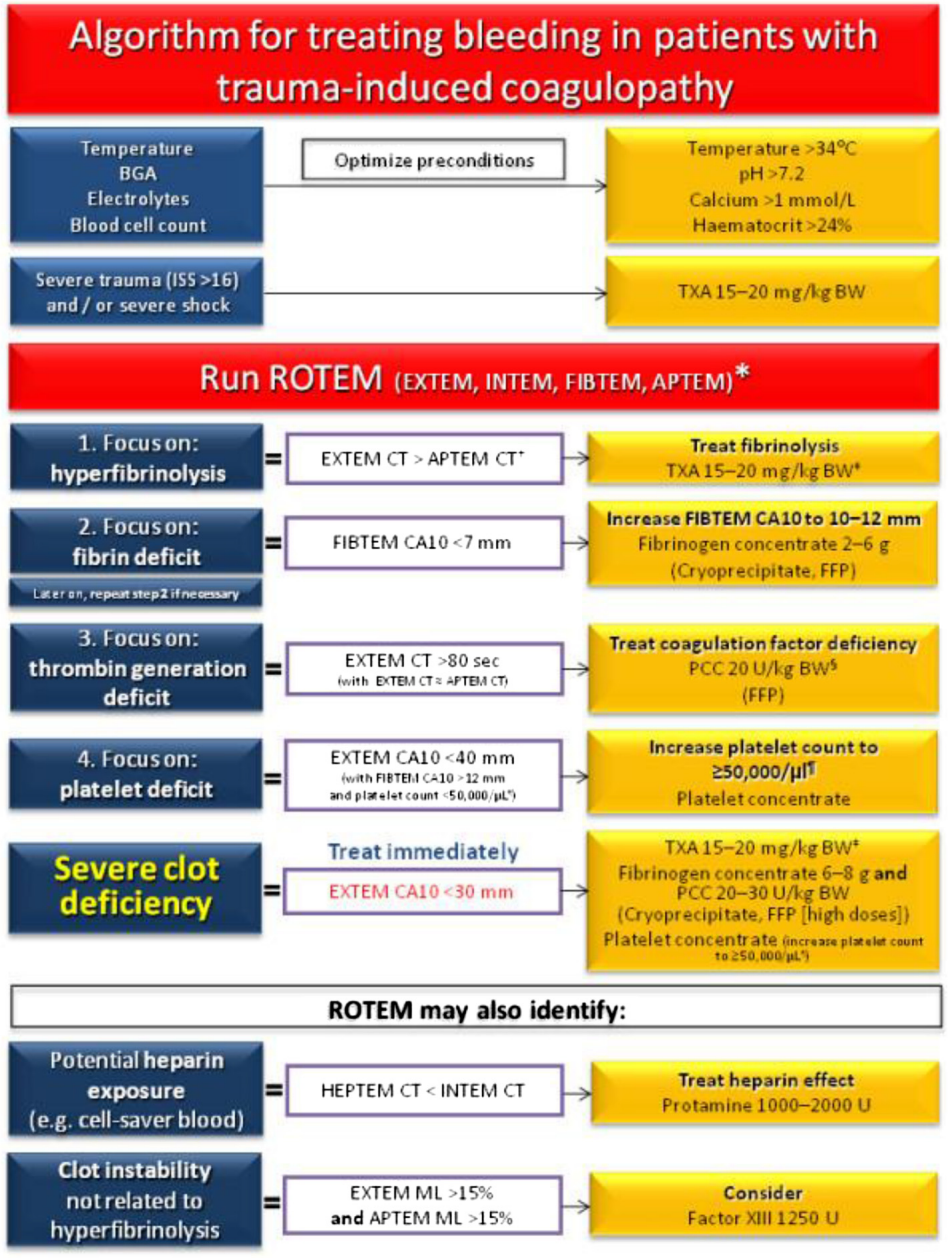
Treatment algorithm of the Cologne-Merheim Medical Center (Germany)

**Fig. 4:**
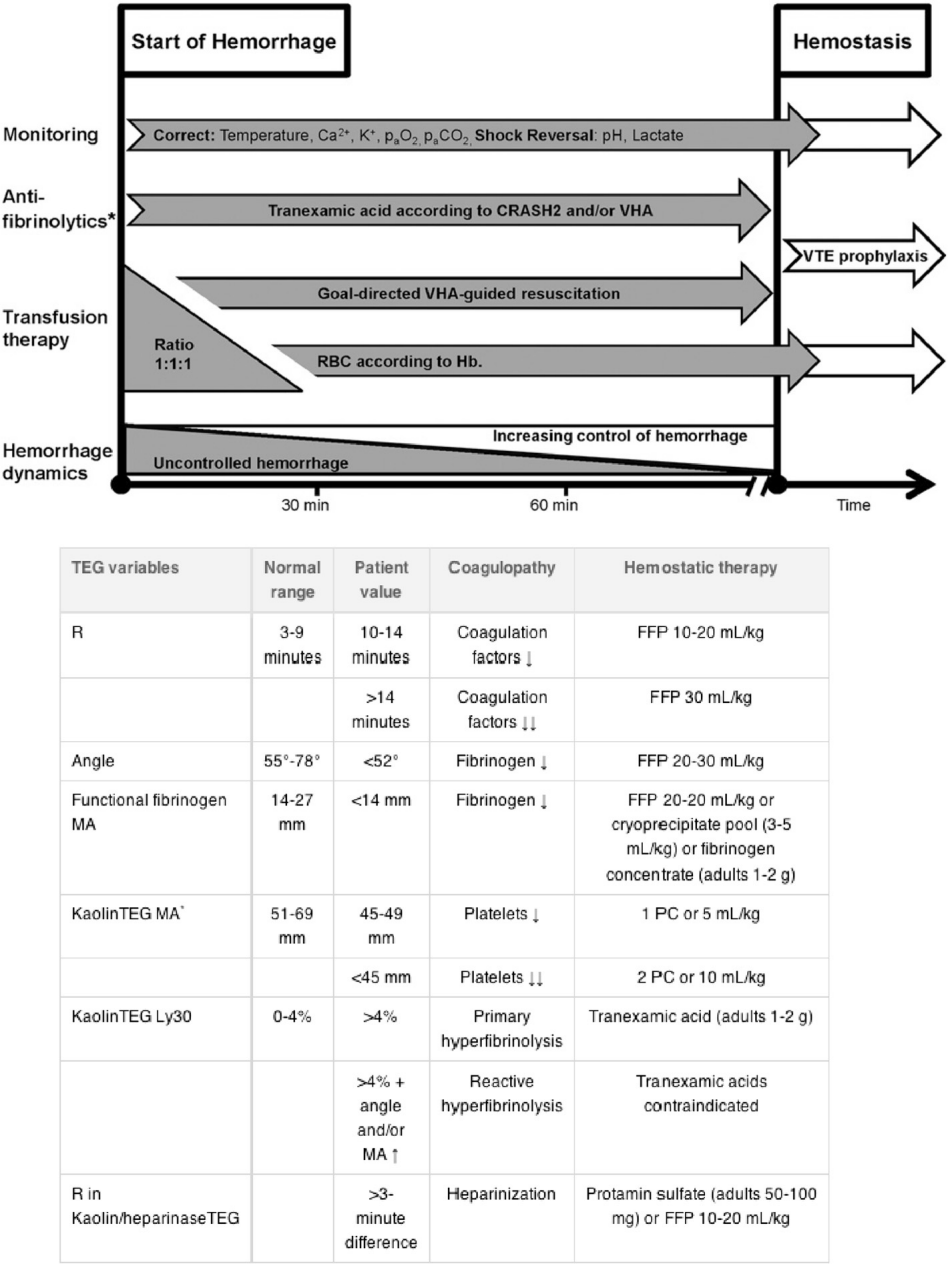
Treatment algorithm of the Rigshospitalet Copenhagen (Denmark)

**Fig. 5:**
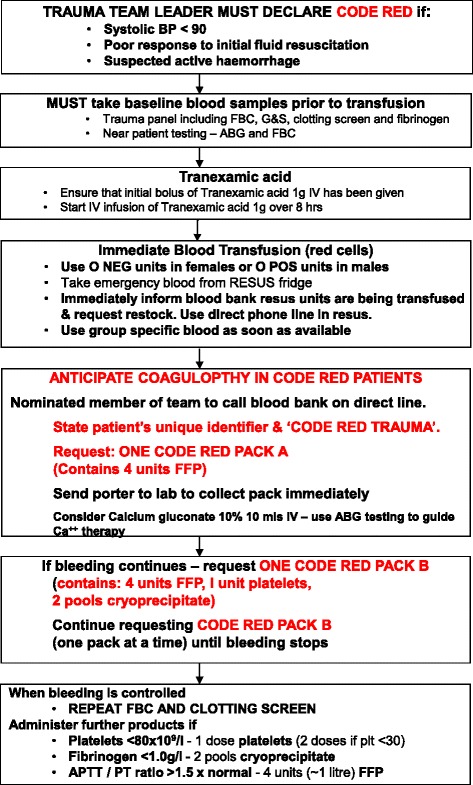
“Code Red” treatment algorithm of the Royal London Hospital (UK)

**Fig. 6:**
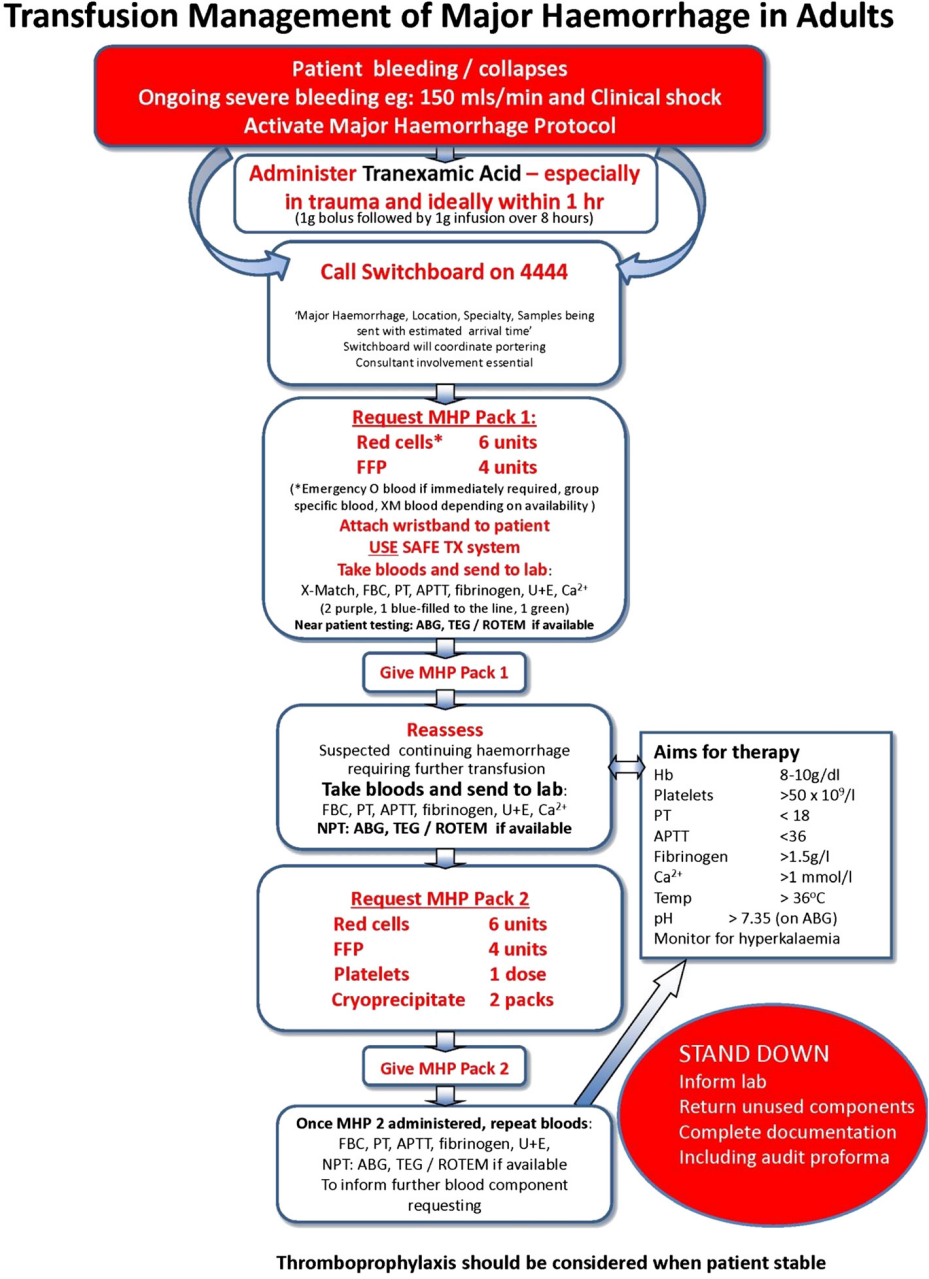
Transfusion management protocol of the John Radcliffe Hospital in Oxford (UK)

The different treatment algorithms and massive transfusion protocols (MTPs) locally in use are shown in Figs. 3, 4, 5 and 6 and Additional files 2 and 3. Although differently structured, the algorithms and protocols in use may be divided into detailed proceeding and controlling steps, defined transfusion packages and/or viscoelastic test-driven actions and therapeutic aims.

#### Activation of the algorithms/protocols

The algorithms and protocols are mostly activated according to clinical assessment indicating (suspected) active/uncontrolled haemorrhage and shock/hypoperfusion combined with poor responsiveness to initial fluid resuscitation and a continuous arterial blood pressure of below 90 mmHg. The immediate administration of tranexamic acid (TXA) in cases of severe trauma and/or shock prior to any other measure or action is a principle component within all algorithms and protocols provided. The first blood samples are analysed for full blood count including platelets, standard coagulation parameters (PT and aPTT), fibrinogen and cross matching. Extended viscoelastic testing on a routine basis is performed in two centres (COL and CPH), and upon availability in other centres (OXF, AMC).

#### Emergency transfusion

All centres provide O Rhesus negative blood units for immediate blood transfusion and switch over to group specific blood bags as soon as cross typing is performed. All algorithms provided address environmental factors further contributing and augmenting coagulopathy and shock, such as hypothermia, acidosis, hypovolaemia and hypocalcaemia. Likewise, all algorithms address antagonism of anticoagulants such as vitamin K and heparin, if intake is known or suspected.

#### Transfusion packs and ratios

Four centres (RLH, OXF, AMC, and OSL; Figs. 5 and 6 Additional files 2 and 3) work with fixed transfusion packages delivered by the local blood banks upon algorithm/protocol activation with blood products aimed to be administered in specified ratios (Table 4). The packages differ between centres with regard to their blood product components and three centres distinguish between two types of packages with second packs (pack B) continuously administered in cases of ongoing bleeding (Table 4). Apart from the first packs, which generally contain standard universal blood products, the second packs also usually contain coagulation factor concentrates according to local availability, for example fibrinogen or cryoprecipitate. A clinical and laboratory reassessment is usually scheduled after each pack administered or at pre-specified time points (OSL with blood gas analysis every 30 min and standard coagulation tests every hour until the bleeding has stopped).

**Table 4:** Transfusion packages per centre

	Amsterdam	Cologne	Copenhagen	London	Oslo	Oxford
Pack A	6 × RBC	4 × RBC	5 × RBC	RBCs	5 × RBC	6 × RBC
	6 × FFP	4 × FFP	5 × FFP	4 × FFP	5 × Octaplas	4 × FFP
	2 × 5 Platelets		2 × Platelets		1 × Platelets	
Pack B	2 g Fibrinogen			4 × FFP		6 × RBC
	1 × 5 Platelets			1 × Platelets		4 × FFP
	100 mg/kg FVIIa			2 × Cryo		1 × Platelets
						2 × Cryo

#### Viscoelastic test-guided algorithms in COL and CPH

Two centres (COL and CPH; Figs. 3 and 4) initiate a transfusion package-based therapy with red blood cell concentrates (RBCs), fresh frozen plasma (FFP) and platelet concentrates (PTLs) (COL with RBC:FFP 1:1 and CPH with RBC:FFP:PLTs 1:1:1 (each PTL comprise a pool of PLT from four donors)) during the initial phase of massive bleeding and perform viscoelastic testing (either thromboelastometry (ROTEM; COL) or thrombelastography (TEG; CPH)) upon arrival of the patient to the trauma bay allowing for an early shift towards a goal-directed viscoelastic test-guided therapy subsequently. Blood samples for viscoelastic testing, blood type and screen are obtained immediately upon arrival to the trauma bay so these patients only receive one transfusion package with universal blood products maximum. Viscoelastic tests are repeated regularly depending upon the dynamics of the bleeding and for every package administered. Hereby, restoration of haemostasis by using adjusted doses of TXA, fibrinogen, prothrombin complex concentrates, platelets, cryoprecipitate and FFP is instituted according to the patient’s individual viscoelastic profile.

#### Target values and end points of resuscitation

It is of utmost importance that algorithms and protocols are discontinued in a timely manner, given the inherent risk of circulatory overload resulting from rapid infusion systems and the risk of thromboembolic events if overtreated with blood products and haemostatic agents to a hypercoagulable state. The aims of therapy are commonly considered across the centres as follow:

- achievement of haemostasis via clinical assessment
- haemoglobin 8–10 g/dl
- PT/a PTT <1.5 × normal (OXF: PT <18 s, aPTT <36 s; OSL: INR <1.5)
- platelets >50 × 10^9^/l (RLH >80 × 10^9^/l; OSL 100 × 10^9^/l)
- Fibrinogen >1.5 g/l (RLH >1.0 g/l; OSL >2 g/l)

The therapeutic targets if viscoelastic testing is used are depicted in the two algorithms for ROTEM and TEG provided by the centres in Cologne (ROTEM; Fig. 3) and Copenhagen (TEG; Fig. 4) [17, 18]. The aim of this resuscitation regimen is to maintain an almost normal viscoelastic haemostatic assay (VHA) profile during the resuscitation phase.

## Discussion

The present study highlights the diversity in clinical practice management of severe trauma haemorrhage. All six trauma centres assessed here have developed and implemented local algorithms and protocols for the bleeding trauma patient and work with multidisciplinary teams within their trauma bays as suggested by the up-dated 2013 European guideline for the management of bleeding and coagulopathy after major trauma [2]. Therefore, the value of treatment algorithms in improving trauma care but also deviations from these pathways has been recognized [3, 4]. Within all settings, these algorithms and protocols are uniformly activated by clinical triggers and deactivated once the bleeding has clinically stopped combined with laboratory signs of achieved haemostasis. In particular the latter is of utmost importance given the potential risks of overtransfusion and overcorrection of haemostasis. Differences among the centres with respect to laboratory targets include the level of fibrinogen and platelets.

The degree of coagulopathy and shock is mostly assessed via standard coagulation tests and ABGs. Of interest, only four centres (only COL and CPH on a regular basis) perform extended viscoelastic testing to assess the severity of the coagulopathy in more detail. Early variables of clot firmness assessed via viscoelastic methods have been demonstrated to be good predictors for the need of massive transfusion and outcome with faster availability compared to standard coagulation tests [17, 18]. The use of viscoelastic methods to assist in characterizing the coagulopathy and in guiding haemostatic therapy is emphasized by the updated European guideline and the grade of recommendation has been lifted from grade 2C in 2010 to grade 1C in 2013 [2].

All centres recognize the immediate use of fibrinolysis inhibitor TXA in cases of severe trauma and/or shock prior to any other measure or even blood sampling based upon the results of the CRASH-2 trial [19]. In order to provide optimum conditions for effective coagulation environmental factors are addressed by all algorithms and protocols [20]. All centres start their initial resuscitation by using transfusion packages with pre-fixed universal blood product combinations immediately delivered to the trauma bay by local blood banks. This approach follows the concept of “damage control resuscitation” by applying blood products at different pre-defined ratios to the bleeding trauma patient [21–23]. While this concept for bleeding trauma patients continues to be implemented and executed around the world, some concerns about using this approach have recently been raised, as it may not achieve correction of either hypoperfusion or coagulopathy during the acute phase of trauma haemorrhage [24, 25]. The recently published PROPPR-trial did not show a difference in mortality at 24 h and 30 days among patients with severe trauma and massive bleeding transfused with plasma, platelets and RBCs in a 1:1:1 compared to a 1:1:2 ratio [26]. If patients are overtriaged to this concept it may also be associated with worse outcomes [27]. As observed in the present study, transfusion packages including applied ratios, although with a common therapeutic aim of stopping bleeding and correcting coagulopathy, may substantially vary between centres with regard to their composition thus making outcome comparisons rather difficult. One avenue to provide a solution would be further evidence in treatment of bleeding patients and - if applicable - an adjustment of protocols [28]. Therefore, delivering universal guidelines for different environments targeting the treatment of haemostatic abnormalities in bleeding trauma patients which are based on primary research and evidence is the aim of the EU FP-7 funded TACTIC project.

Two centres (COL and CPH) initially start with transfusion packages but with viscoelastic tests running in parallel to quickly allow to shift towards a viscoelastic test-guided therapy subsequently [29, 30]. Via this approach the patients receive one unguided transfusion package with universal blood products at maximum in the acute phase and may thus be prevented from overtransfusion and the potentially harmful effects of allogenic blood products [31–33]. The overall and targeted aim of this approach is to maintain as normal viscoelastic profiles as possible during the resuscitation phase, given that hypocoagulable profiles have repeatedly been associated with increased mortality [30, 34].

The optimum way to resuscitate bleeding trauma patients remains unclear and, as shown in the present study, diversity in clinical practice management exists even across major level I centres. Although it may be likely that goal-directed viscoelastic test-guided haemostatic resuscitation is superior over unguided administration of transfusion packages aiming for the administration of pre-fixed ratios of universal blood products, data from prospective, randomized clinical trials that support either approach are still lacking. The authors expect to start such a trial in 2015 in context of the recently EU FP-7 funded TACTIC project which involves the six trauma centres also involved in the present study.

Due to the small number of centres participating the results of this study should be regarded as expert opinion, which therefore is limiting the expressiveness of the findings presented.

## Conclusions

This study offers valuable information about similarities and differences in the acute treatment of bleeding patients in six different trauma centres across central and northern Europe. With regard to limitations of this analysis of a questionnaire and different transfusion protocols accordingly, the results presented show that in all centres algorithms for the acute treatment do exist. Although recommended in the updated European guidelines even in the participating centres viscoelastic guided therapy and pre-hospital blood administration cannot be regarded as standard approach.

Thus, this study underlines the importance of further primary research followed by clinical trials to improve the evidence for sophisticated guidelines, which is a future aim of the EU FP-7 TACTIC project.

## Additional files

Additional file 1:
**Online survey consisting of 13 questions to query responsibilities, strategies and availabilities in the demanding treatment of trauma patients.** Single answer choices are represented as circles and multiple answer options as boxes. (DOCX 85 kb) Additional file 2:
**Massive transfusion protocol of Academic Medical Centrum, Amsterdam (The Netherlands).** (PPTX 386 kb) Additional file 3:
**Massive bleeding protocol of the Oslo University Hospital Ulleval (Norway).** (PPTX 409 kb)
