# Supplementary material for: Diversity in clinical management and protocols for the treatment of major bleeding trauma patients across European level I Trauma Centres
Source: Scand J Trauma Resusc Emerg Med. 2015 Oct 1;23:74. doi: 10.1186/s13049-015-0147-6 (PMC4590713; doi:10.1186/s13049-015-0147-6)
Supplement: Additional file 1: — Online survey consisting of 13 questions to query responsibilities, strategies and availabilities in the demanding treatment of trauma patients. Single answer choices are represented as circles and multiple answer options as boxes. (DOCX 85 kb) [file 13049_2015_147_MOESM1_ESM.docx]

**Supplemental S1**

1. Please choose only one of the following:


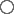
 Amsterdam, the Netherlands


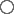
 Cologne, Germany


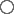
 Copenhagen, Denmark


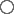
 London, UK


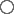
 Oslo, Norway


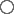
 Oxford, UK

2. What is the approximate number of severely injured trauma patients (Injury Severity Score (ISS) ≥16) admitted to your hospital/trauma centre per year?

Please choose only one of the following:


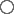
 < 100


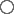
 101 - 200


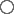
 201 - 300


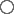
 301 - 400


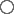
 > 401

3. What is the estimated percentage of bleeding trauma patients with coagulopathy and need for haemostatic therapy in your Hospital / Trauma Centre?

Please choose only one of the following:


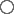
 < 10%


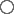
 11 - 30%


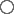
 31 - 50%


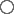
 51 - 80%

4. Who in your institution is primarily responsible for the initial management of bleeding trauma patients including coagulation management?

(Multiple answers possible if more than one speciality (multidisciplinary team))

Please choose all that apply:


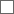
 General Surgery


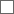
 Trauma / Orthopedic Surgery


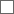
 Vascular Surgery


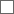
 Neurosurgery


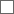
 Anaesthesiology


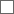
 Transfusion Medicine


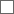
 Haematology


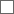
 Intensive Care / Critical Care Medicine


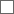
 Accident and Emergency Medicine


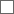
 General Medicine


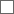
 Other:

5. What are the strategies/parameters followed in your institution to (rapidly) assess, manage and monitor haemostatic disorders / coagulopathy after trauma?

Please choose the appropriate response for each item:

|  | in use | not used |
| --- | --- | --- |
| Haemoglobin (Hb) | 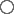 | 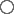 |
| Haematocrit (Hct) | 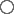 | 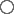 |
| PT/INR/Quick | 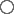 | 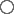 |
| aPTT | 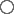 | 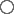 |
| Platelet count (plts) | 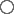 | 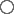 |
| Platelet function (e.g. Aggregometry) | 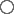 | 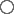 |
| Fibrinogen (quantitative) | 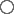 | 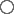 |
| Fibrinogen (functional) | 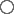 | 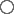 |
| Viscoelastic tests (TEG/ROTEM) | 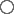 | 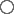 |
| Lactate | 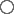 | 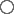 |
| pH | 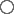 | 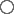 |
| Base excess /deficit (BE/BD) | 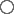 | 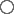 |
| Ionised Calcium | 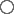 | 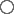 |
| Scoring systems (e.g. TASH) | 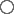 | 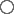 |
| FAST Ultrasound | 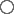 | 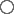 |
| Imaging (CT) | 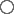 | 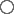 |
| Peritoneal Lavage | 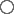 | 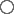 |

6. What are the usual turn-around times for the coagulation assays used in your institution?

Please choose the appropriate response for each item:

|  | <30 min | 31 - 60min | >60min | not used |
| --- | --- | --- | --- | --- |
| Haemoglobin (Hb) | 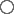 | 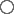 | 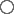 | 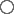 |
| Haematocrit (Hct) | 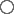 | 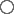 | 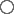 | 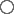 |
| PT/INR/Quick | 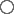 | 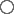 | 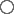 | 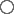 |
| aPTT | 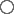 | 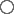 | 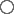 | 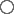 |
| Platelet count (plts) | 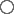 | 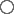 | 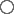 | 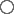 |
| Platelet function (e.g. Aggregometry) | 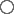 | 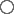 | 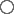 | 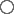 |
| Fibrinogen (quantitative) | 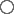 | 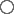 | 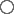 | 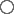 |
| Fibrinogen (functional) | 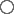 | 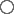 | 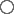 | 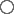 |
| Viscoelastic tests (TEG/ROTEM) | 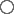 | 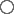 | 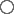 | 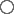 |
| Lactate | 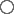 | 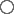 | 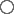 | 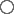 |
| pH |  |  |  |  |
| Base excess /deficit (BE/BD) |  |  |  |  |
| Ionised Calcium |  |  |  |  |

7. What types of blood products to support coagulation function are available in your institution for the management of bleeding trauma patients?

Please choose all that apply:

Packed red blood cell concentrates (pRBCs)

Fresh whole blood (WB)

Fresh frozen plasma concentrates (FFPs)

Thawed fresh plasma

Lyophilized plasma (LP) / Freeze-dried plasma

Platelet concentrates (single platelet units or aphaeresis packs)

Fibrinogen concentrate

Single factor concentrate (rFVIIa)

Single factor concentrate (FXIII)

Single factor concentrate (other)

Cryoprecipitate (FVIII, Fibrinogen, vWF, FXIII)

Prothrombin complex concentrate (3- or 4-factor PCC concentrates; PPSB)

(FII;(FVII);FIX;FX,protein C and S)

Other:

8. What types of supplementary agents / drugs to support coagulation function are available in your institution for the management of bleeding trauma patients?

Please choose all that apply:

Tranexamic acid (TXA)

Aminocaproic acid

Calcium (Ca++)

Desmopressin

Vitamin K

Albumin

Other:

9. What is the realistic time frame between arrival of the bleeding trauma patient in the Emergency Department (ED) and the administration of the first blood product in your institution?

Please choose all that apply:

< 15min

16 - 30min

31 - 60min

> 60min

pre-hospital administration of blood products possible

10. What are the issues addressed by the treatment algorithm implemented in your institution for the management of bleeding and coagulopathy after trauma?

a.) Initial Resuscitation

Please choose all that apply:

Time management

Local bleeding control via tourniquets and compression

Mechanical ventilation

Target systolic blood pressure and vasopressor use

Fluid resuscitation with crystalloids

Fluid resuscitation with colloids

Fluid resuscitation with hypertonic solutions

Body temperature (hypothermia)

Other:

b.) Assessment / Investigation / Monitoring

Please choose all that apply:

Clinical assessment of haemorrhage

Early imaging (FAST and/or CT)

Coagulation monitoring via standard coagulation assays (e.g. PT, INR, Quick, aPTT,

Fibrinogen, Platelet count etc.)

Advanced coagulation monitoring (e.g. viscoelastic tests, aggregometry, multiplate, platelet

mapping etc.)

Acidosis

Other:

c.) Immediate Intervention

Please choose all that apply:

Damage control strategies (Emergency Surgery)

Local haemostatic procedures

Use of packed red blood cell concentrates (pRBCs)

Use of fresh frozen plasma concentrates (FFP)

Use of platelet concentrates

Use of blood products (pRBCs, FFPs, platelets) in ratios

Use of coagulation factor concentrates

Use of antifibrinolytics (e.g. ε-tranexamic acid (TXA))

Use of calcium

Other:

In case of a ratio-based transfusion which ratio of pRBCs:FFPs:Plts is applied?

Please write your answer here:
