# Supplementary figures and images for: Diversity in clinical management and protocols for the treatment of major bleeding trauma patients across European level I Trauma Centres
Source: Scand J Trauma Resusc Emerg Med. 2015 Oct 1;23:74. doi: 10.1186/s13049-015-0147-6 (PMC4590713; doi:10.1186/s13049-015-0147-6)

## Slide 1
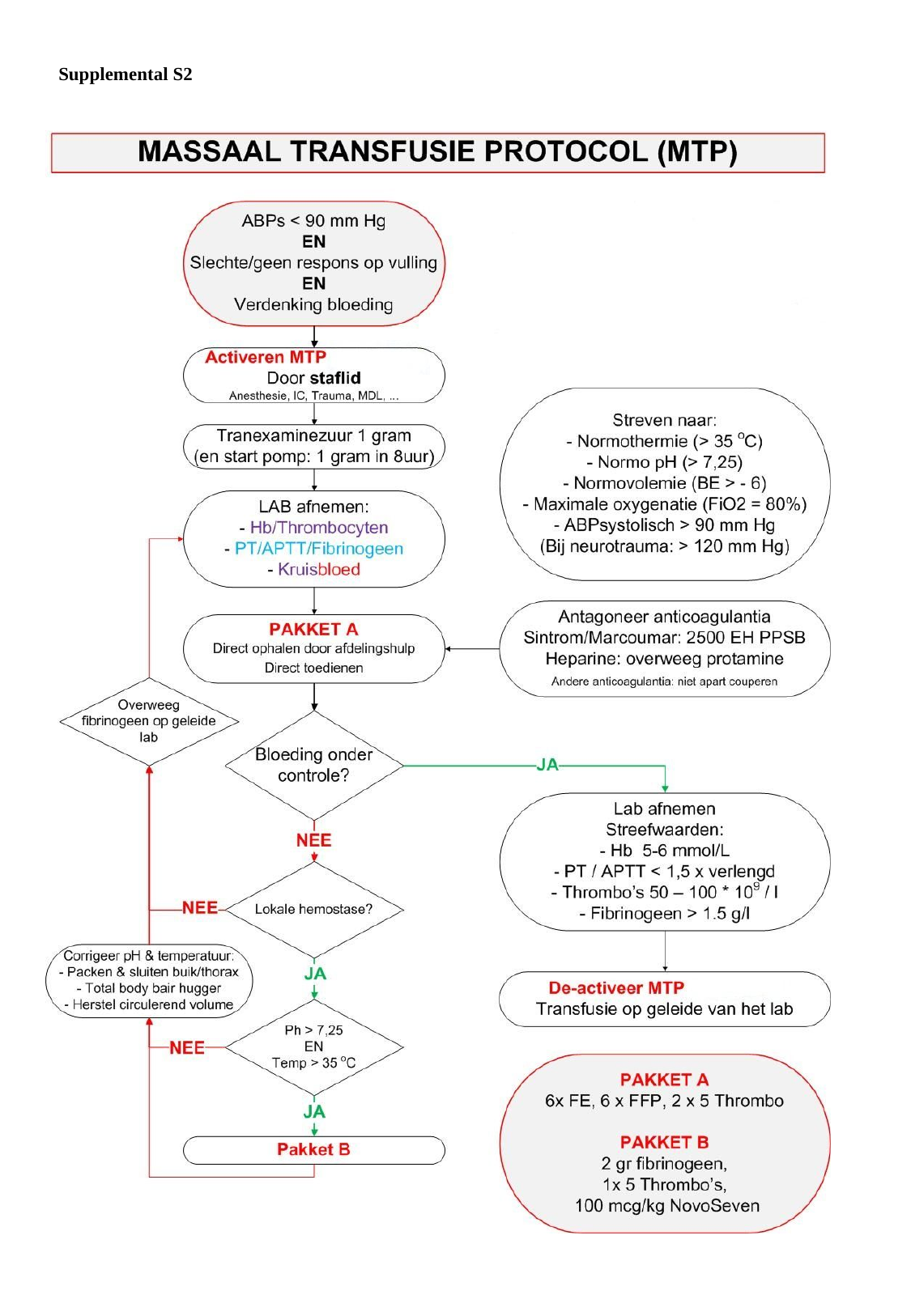

Supplemental S2

Supplement: Additional file 2: — Massive transfusion protocol of Academic Medical Centrum, Amsterdam (The Netherlands). (PPTX 386 kb) [file 13049_2015_147_MOESM2_ESM.pptx]

## Slide 1
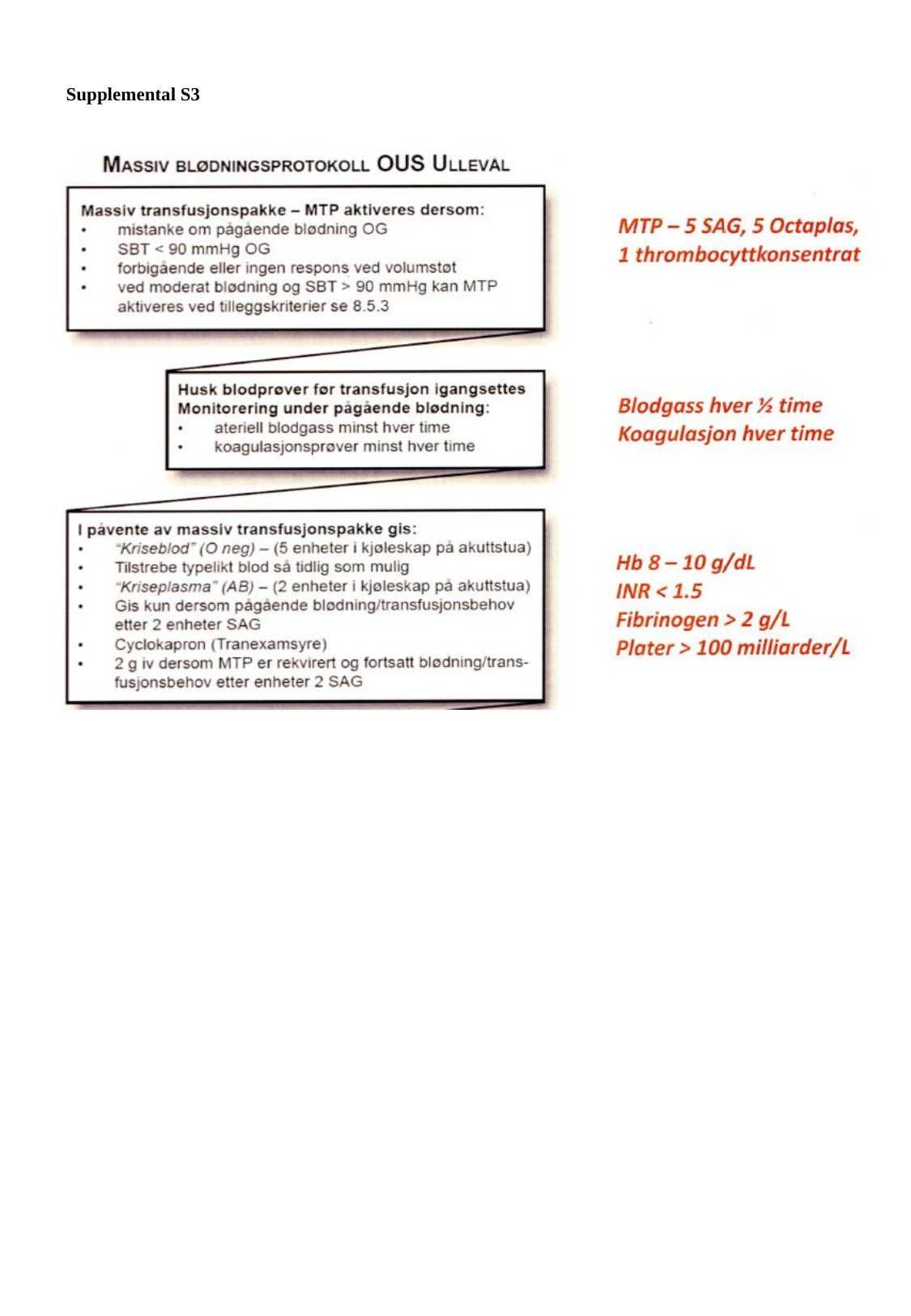

Supplemental S3

Supplement: Additional file 3: — Massive bleeding protocol of the Oslo University Hospital Ulleval (Norway). (PPTX 409 kb) [file 13049_2015_147_MOESM3_ESM.pptx]
